# Apoptosis markers of circulating leukocytes are associated with the clinical course of inflammatory bowel disease 

**Published:** 2018

**Authors:** Azade Amini Kadijani, Fariba Javadinia, Alireza Mirzaei, Zeinab Khazaei koohpar, Hedieh Balaii, Shaghayegh Baradaran Ghavami, Zahra Gholamrezaei, Hamid Asadzadeh-Aghdaei

**Affiliations:** 1 *Gastroenterology and Liver Diseases Research Center, Research Institute for Gastroenterology and Liver Diseases, Shahid Beheshti University of Medical Sciences, Tehran, Iran*; 2 *Basic and Molecular Epidemiology of Gastrointestinal Disorders Research Center, Research Institute for Gastroenterology and Liver Diseases, Shahid Beheshti University of Medical Sciences, Tehran, Iran*; 3 *Bone and Joint Reconstruction Research Center, Shafa Orthopedic Hospital, Iran University of Medical Sciences, Tehran, Iran*; 4 *Assistant Professor, PhD in Cellular and Molecular Biology, Department of Cellular and Molecular Biology, Faculty of Biological Sciences, Tonekabon Branch, Islamic Azad University, Tonekabon, Iran *

**Keywords:** Inflammatory bowel diseases, Apoptosis, Remission, Flare-up, Biomarker.

## Abstract

**Aim:**

Here we aimed at evaluating whether the apoptosis status of circulating leukocytes of inflammatory bowel diseases (IBD) patients is attributed to the diseases clinical status.

**Background:**

Defects in the programmed cell death of inflammatory cells is known as to play a major role in the pathogenesis of IBD, and has been associated with the clinical efficacy of therapeutic agents.

**Methods:**

A total of 50 IBD patients, 25 with remission and 25 with flare-up phase of the disease, who their disease was confirmed by colonoscopy, were included in this cross-sectional study. Pro-apoptotic Bax and anti-apoptotic Bcl-2 mRNA expression, along with Bax/Bcl-2 ratio, as measures of apoptotic status, were assessed in the Peripheral blood mononuclear cell (PBMC) of the patients using semi-quantitative Real-time PCR method.

**Results:**

The mean Bax mRNA expression level was 0.54±0.12 in flare-up group and 0.53±0.13 in remission group (p=0.8). The mean Bcl-2 mRNA expression level was 0.63±0.13 in flare-up group and 0.55±0.12 in remission group (p=0.03). The mean Bax/Bcl-2 ratio was 0.88±0.17 in flare-up group and 1±21 in remission group (p=0.05). The mean Bax/Bcl-2 ratio was not statistically significant between different disease types (p=0.54) or therapeutic agents (p=0.7).

**Conclusion:**

According to our results, alteration in markers of apoptosis could be traced in the circulating leukocytes of IBD patients, which suggest a potential for clinical application of apoptosis markers in disease monitoring and prediction of relapse.

## Introduction

 Inflammatory bowel diseases (IBD), including ulcerative colitis (UC) and Crohn’s disease (CD), are worldwide health-care problem characterized by periods of relapse (flare-up) and clinical remission (1-3). Although the etiology of IBD remains largely unknown, pathogenesis of IBD is partially understood (4). In this respect, the role of intrinsic defects in the control of programmed cell death of the mucosal T cells of IBD patients has been unraveled (5). While the normal intestinal T cells (ITCs) show an increased susceptibility towards apoptosis following the stimulation by a specific antigen, the ITCs of IBD patients are resistant to apoptosis, showing a prolonged survival along with an increased cytokine production that significantly aggravates the inflammation (6, 7).

According to this evidence, the clinical efficacy of therapeutic agents has been associated with the induction of apoptosis in activated leukocytes of IBD patients (8, 9). If that were the case, an enhanced apoptosis rate could be expected in circulating leukocytes of IBD patients who respond to therapy (patients in remission) compared to those who do not (patients in flare-up). 

In spite of the existing evidence on the main clinical, genetic, endoscopic, histologic, serologic and fecal markers to predict the clinical course of IBD, no single marker seems to be reliable enough to predict the disease relapse. Thus, more reliable prognostic markers need to be elaborated (10).

Here we evaluate the Bax, Bcl-2 and Bax/Bcl-2 ratio, as markers of the apoptotic status of circulating leukocytes of IBD patients, in order to explore if any of these markers are able to distinguish remission from relapse in IBD patients. 

## Methods

In this cross-sectional study, 50 IBD patients, who their disease was confirmed by colonoscopy, were included. Treatment strategy consisted of aminosalicylates as the first therapeutic choice and corticosteroids and biologics as the later choices (11). Clinical and endoscopic evaluation of the disease phase was performed by one specialist. In this regard, Crohn's Disease Endoscopic Index of Severity (CDEIS) and Mayo score was used to define disease severity in CD and UC, respectively (12, 13). 

Patients with evidence of systemic infections and patients suffering from other concomitant immunological disorders were excluded from the study.

A total of 10 ml Blood samples were taken from each patient. Peripheral blood mononuclear cell (PBMC) were extracted by gradient centrifugation on a layer of Ficoll-Paque Plus (Amersham, Uppsala, Sweden) and preserved at -80°C for later examinations. 

**Figure 1:**
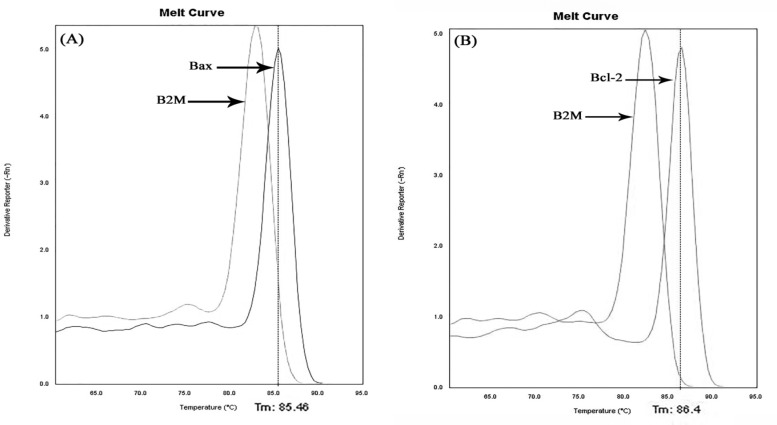
Melting curves of Real-time PCR product for (A) Bax and (B) Bcl-2 gene

Pro-apoptotic Bax and anti-apoptotic Bcl-2 mRNA expression, along with Bax/Bcl-2 ratio, as measures of the apoptotic status of the PBMC, were evaluated using semi-quantitative Real-time PCR method. In this respect, after the RNA extraction using YTA RNA Extraction kit (YektaTajhizAzma, Tehran, Iran), complementary DNA (cDNA) was synthesized by Revert Aid First Strand cDNA Synthesis Kit (Thermo Scientific, USA). Real-time PCR was performed afterward using SYBR® Premix Ex Taq™ II (Takara, Japan) on a StepOnePlus™ Real-Time PCR System (Applied Biosystems, USA) for 1 cycle at 95 °C for 2 min followed by 40 cycles at 95 °C for 5s and 60 °C for 30s. Specificity of each product was verified by the analysis of melting curve (Fig.1). Finally, data were analyzed using comparative Ct method, in which 2^−ΔCt ^where ΔCt is (CT gene of interest−CT internal control) (14) was computed for each sample and used for statistical analysis. REST 2009 software was used for gene expression analysis using real-time amplification data.

β-2 microglobulin (B2M) was used as internal control. The sequences of primers were as follows: B2M F: TGC TGT CTC CAT GTT TGA TGT ATCT, B2M R: TCT CTG CTC CCC ACC TCT AAGT. Bax F: CCT GTG CAC CAA GGT GCC GGA ACT, Bax R: CCA CCC TGG TCT TGG ATC CAG CCC. Bcl-2 F: TTG TGG CCT TCT TTG AGT TCG GTG, Bcl-2 R: GGT GCC GGT TCA GGT ACT CAG TCA. 

This study has been approved by the institutional review board of our university and informed consent has been obtained from each patient in order to enroll in the study.

**Statistical analysis:**

The data were analyzed using IBM SPSS for Windows, version 16. Descriptive variables were demonstrated by the mean and standard deviation (SD). Independent T-test or Mann-Whitney U test was used for the evaluation of the mean deference of variables between remission and flare-up group. A p value of less than 0.05 was regarded as statistically significant. 

## Results

In total, 25 patients with flare-up phase and 25 patients with remission phase of disease were identified, which included 31 females and 19 males with the mean age of 37.4±13 years. The patient population consisted of 42 UC and 7 CD patients. In one patient the disease type was missing. Regarding the medication history, 14 patients have received aminosalicylates alone. Aminosalicylates and corticosteroids were used in 16 patients. Aminosalicylates, corticosteroids, and Infliximab were used in 10 patients. Medication history was not available for the other 10 patients. The clinical and demographic characteristics of patients with respect to the disease course have been summarized in Table 1.

**Table 1:** Demographic and clinical characteristics of IBD patients according to the phase of the disease

Variable	Remission group (n=25)	Flare-up group (n=25)	P value
Age (years)	38.3±14	36.6±12.1	0.7
Gender			
Female Male	64 36	60 40	0.77
Family history			
Negative Positive Missing	19 (76) 1 (4) 5 (20)	21 (84) 0 (0) 4 (16)	0.31
Disease Type			
UC CD Missing	22 (88) 2 (8) 1 (4)	20 (80) 5 (20) 0	0.24
Therapeutic agent			
Aminosalicylates Corticosteroids Biologics Missing	8 (32) 7 (28) 5 (20) 5 (20)	6 (24) 9 (36) 5 (20) 5 (20)	0.53

Data are shown as number(%) or mean±SD. P<0.05 is regarded significant.

**Figure 2:**
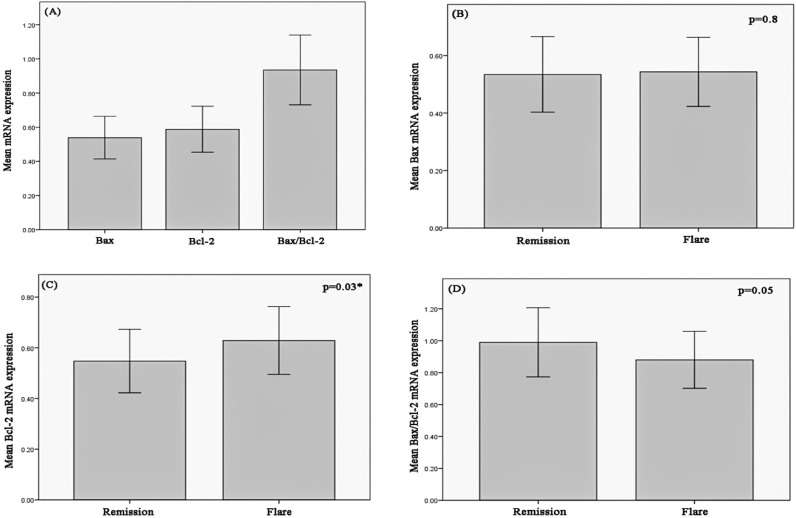
(A) Real time PCR analysis showing the mean Bax, Bcl-2, and Bax/Bcl-2 mRNA expression levels; (B) Comparison of mean Bax mRNA expression between remission and flare-up phases of IBD; (C) Comparison of mean Bcl-2 mRNA expression between remission and flare-up phases of IBD; (D) Comparison of mean Bax/Bcl-2 mRNA expression between remission and flare-up phases of IBD; Data are shown as mean±SD. (*) shows a significant p value (<0.05)

The mean Bax mRNA expression level was 0.54±0.12, ranging from 0.3 to 0.8. The mean Bcl-2 mRNA expression level 0.59±13, ranging from 0.36 to 0.9. The mean BAX/Bcl-2 ratio was 0.94±0.2, ranging from 0.0.56 to 1.44 (Fig.2A). The mean Bax mRNA expression level was 0.54±0.12 in flare-up group and 0.53±0.13 in remission group. This small difference was not statistically significant (p=0.8) (Fig.2B). The mean Bcl-2 mRNA expression level was 0.63±0.13 in flare-up group and 0.55±0.12 in remission group. This difference was significant (p=0.03) (Fig.2C). The mean Bax/Bcl-2 ratio was 0.88±0.17 in flare-up group and 1±21 in remission group. This difference was marginally significant (p=0.05) (Fig.2D).

The mean Bax/Bcl-2 ratio was 0.95±0.21 in UC and 0.9±0.13 in CD patients. This difference was not statistically significant (p=0.54). Moreover, the mean Bax/Bcl-2 ratio was not significantly different in patients receiving various therapeutic agents (p=0.7). In this respect, the mean Bax/Bcl-2 ratio was 0.87±0.11, 0.94±0.24, and 0.95±0.22 in patients receiving aminosalicylates, corticosteroids, and biologics, respectively. No significant association was also observed between Bax/Bcl-2 ratio and demographic characteristics of patients such as age and gender.

## Discussion

Apoptosis plays an essential role in immune system regulation through the depletion of expanded effector T cells following the immune responses. Impaired apoptosis results in several dysfunctions in the immune system such as autoimmunity (15).

A dysregulated apoptosis is known to play a role in the pathogenesis of IBD, so that an excessive intestinal cell shedding and barrier loss in the remission phase of IBD predicts a disease relapse (16). In addition, a variety of apoptotic bodies were found in colonic biopsies routinely taken from UC patients with active disease, suggesting an association between intestinal epithelial cell (IEC) apoptosis and disease severity (17). Elevated apoptosis was also observed in colonic samples of CD patients compared to controls (18). 

By contrast, intestinal T cells (ITC) of IBD patients were found to be resistant to several apoptotic signals, suggesting a possible mechanism to describe why inflammation is refractory to resolution in IBD patients (15).

Based on the available evidence, apoptosis of effector inflammatory cells has been attributed to the clinical efficacy of IBD therapeutic agents in several investigations (8, 19-21). 

Sabatino *et al. *evaluated apoptosis-inducing potential of IFX in lamina propria T cells (LPT) of CD patients before and after three consecutive infusions of IFX. Based on their report, IFX treatment induced a sustained apoptosis in LPT. Furthermore, IFX treatment induced apoptosis in circulating T cells isolated from CD patients, although to a lesser extent compared to the LPT (22). 

Similar investigations have also shown induced apoptosis in activated peripheral blood leukocytes of IBD patients following the treatment (23, 24). Moreover, different sensitivity to apoptosis following the treatment of patient’s PBMC with anti-tumor necrosis factor-alpha has been reported in the study of Coury*et al. *(25). Accordingly, we hypothesized that a higher apoptosis could be induced in the circulating leukocytes of IBD patients who respond to the therapy and aimed to evaluate this hypothesis. 

In this study, we compared Bax, Bcl-2, and Bax/Bcl-2 ratio between remission and flare phase of IBD. Our results showed a significantly lower Bcl-2 mRNA expression and higher Bax/Bcl-2 ratio in the PBMC of remission group compared to the flared-up patients. 

Amini *et al.* in a prospective study evaluated the apoptosis-inducing potential of Infliximab (IFX) in the peripheral blood leukocytes of IBD patients. According to their results, apoptosis rate was significantly higher in circulating leukocytes of IFX responders compared to IFX non-responders (19). In another attempt, this group evaluated the association of response to IFX with Bax/Bcl-2 ratio in the PBMC of IBD patients. They found a direct association between the Bax/Bcl-2 ratio and therapeutic response to IFX (9). We also found a direct association between the Bax/Bcl-2 ratio and clinical phase of IBD. 

In a similar study, El-Hodhod*et al*. evaluated the apoptosis status of peripheral lymphocyte during IBD flare and remission. Their results showed enhanced blood lymphocytes apoptosis in children with IBD compared. To our surprise, their results also showed significantly higher early apoptotic indices in the blood lymphocytes of flare-up group compared to remission group, which was inconsistent with our results. However, it should be remembered that the method of evaluation of apoptosis was different in their study (26).

Taken together, the existing evidence suggests that apoptosis-inducing potential of therapeutic agents could also be traced in the blood circulation of IBD patients, and could be used for prognostic or monitoring purposes.

Our study has some weaknesses which should be pointed out. The small sample population could be regarded as the biggest limitations of the study. Thus we suggest future investigations with larger sample size in order to shed more light on the apoptosis status of circulating leukocytes of IBD patients and its clinical implications.

In conclusion, alteration in apoptosis markers could be traced in the circulating leukocytes of IBD patients and has the potential to distinguish IBD remission from flare-up phase. These results further confirm a systemic nature for IBD, and suggest a clinical role for apoptosis status in disease monitoring and prediction of relapse.

## Conflict of interests

The authors declare that they have no conflict of interest.
